# Transient Left-Sided Paralysis following Robotic-Assisted Laparoscopic Uteropexy

**DOI:** 10.1155/2015/150715

**Published:** 2015-05-26

**Authors:** Jasmina Kurdija, Jan G. Jakobsson

**Affiliations:** Department of Anaesthesia & Intensive Care, Institution for Clinical Science, Karolinska Institutet, Danderyds Hospital, 182 88 Stockholm, Sweden

## Abstract

We describe a case report of a 47-year-old ASA 2 female patient who exhibits severe headache and hemineurology during awakening following robotic pelvic prolapse surgery. The symptoms resolved spontaneously during the first postoperative day. We could not find any explicit root cause. Robotic surgery associated adverse events are discussed.

## 1. Introduction

Robotic surgery is increasingly adopted. Robotic technique is increasingly used for urology and gynecoligical surgery [1, 2]. A number of procedures are nowadays conducted with robotic technique improving the surgical outcome. Robotic surgery demands however an adequate anaesthesia strategy. Procedures such as prostatectomy and prolapse surgery demand furthermore that the patient is placed in Trendelenburg position and CO_2_ gas is insufflated creating a surgical field. Gas insufflations have a number of effects reducing venous return, in combination with head-down moving diaphragm, and subsequently impairing ventilation perfusion matching and further increasing intracranial pressure. Vascular gas entrainment causing intravascular gas emboli is also a well-known risk associated with surgery requiring CO_2_ insufflation. Thus robotic surgery is associated with risks and the benefit versus risk must be acknowledged [3]. We describe a patient complaining of severe headache and hemiparesis after awakening from robotic surgery. The symptoms resolved spontaneously during the first 24 postoperative hours. Side effects and complications associated with robotic surgery and CO_2_ insufflation are discussed.

## 2. Case Report

The patient has given informed consent to present this case report. A 47-year-old woman with uterus prolapse was admitted for elective robotic prolapse surgery. The patient had a BMI 32 but was otherwise healthy. She had previously been examined with transthoracic echocardiography (TTE) and 24 h Holter EKG because of subjective symptoms of arrhythmia. Nothing pathologic was found except for sparse supraventricular extrasystoles. She has also had an asthma attack 2 years prior to this. She had no current medication.

She underwent selective standard robotic-assisted laparoscopic uteropexy. The operation was performed in general anaesthesia. Anaesthesia was induced with intravenous propofol 200 mg and target controlled remifentanil infusion. The patient was muscle-relaxed with rocuronium 40 mg and intubated. Anaesthesia was maintained with target controlled remifentanil infusion and sevoflurane. She was monitored with EKG, pulse oximetry, and capnography, and the blood pressure (BP) was monitored with an arterial line in the left radial artery. Immediately after the start of anaesthesia patient's BP dropped to 90–100/50 mmHg and remained that way during the preoperative preparations until start of surgery. During the operation that lasted approximately 2.5 h, the patient was in Trendelenburg position (27 degrees head down). She was respiratory and circulatory stable during the operation with a BP around 115/70 and heart rate of 60–75 beats per minute. SpO_2_ was 99% with FiO_2_ 0.35–0.5, EtCO_2_ 4.3–5.2, and peak pressure in the ventilator was 17–30 cmH_2_O.

During emergence from anaesthesia the patient woke up and was moving all four extremities. She was taken to the postoperative ward where she initially was sleeping. She woke up after two hours with anxiety, severe headache, and complaint of loss of sensation in the left side of the body. She could move her right arm and leg. She was able to do minor movements in left leg and arm but was not able to lift left arm or leg from the surface of the bed. There was a sensory impairment on the left side compared to the right, with lowered sensation for pain and touch. A neurologic examination showed no other abnormities.

The patient underwent an acute computed tomography (CT) of the brain and CT-angiography of the arteries in the neck and brain. Both examinations were normal with no detected haemorrhage, infarction, or expansive process/oedema in the brain. There was no thrombosis, occlusion, or stenosis in the arteries.

The patient was discharged from the recovery room and admitted to a neurologic ward. During the night the patient's symptoms improved and within 24 hours she was fully restored. All symptoms resolved spontaneously, and no interventions were undertaken. All follow-up examinations including MRI of the brain, blood tests, and lumbar puncture were normal. She was discharged home after three days without any neurological sequelae.

We were not able to make conclusive diagnosis for the patient's transient left-sided paralysis. There are several potential causes: positioning injury, gas embolism, migraine, and transient ischemic attack.

## 3. Positioning Injury

There is a known risk for perioperative positioning injuries. With the not uncommonly extreme positioning head-down tilt positioning of patients undergoing robotic-assisted surgery (RAS) that risk may be increased. Some authors report Trendelenburg position of even 45 degrees [4]. Examples of such injuries specific to RAS are compartment syndrome, rhabdomyolysis, ischemic optic neuropathy, and upper or lower extremity peripheral neuropathies [5]. Mild-to-severe stretching or compression of a specific nerve or nerve plexus is thought to be the cause of the nerve damage in these cases. Mills et al. [6] looked specifically at nerve damage associated with robotic-assisted urological surgery and found that the factors significantly associated with injury were long operative time (>328 min), in room time and ASA classes 2–4. Time required to recover from these injuries varies with the severity of the injury [7, 8]. Our patient was positioned on a vacuum mattress containing beans with her arms at the side. Pads for shoulder support were fixated on the operating table. Her legs were put in leg braces so that she could be put in a lithotomy position if needed. She underwent a normal length surgery (146 minutes), in room time being 253 minutes, and is ASA 2. Directly after the finish of surgery she was examined for pressure damage and none was found. Her symptoms are unlikely related to peripheral neuropathy since she experienced paralysis in both her arm and her leg, which together with severe headache suggest a central cause.

## 4. Gas Embolism

Venous gas embolism can cause acute symptoms such as tachycardia, cardiac arrhythmias, hypotension, desaturation, or EKG changes. Studies have been performed using transesophageal echocardiography (TEE) to detect gas embolism in the right atrium. Incidences of embolism with or without cardiorespiratory symptoms have been reported in up to 100% of cases during total laparoscopic hysterectomy, 69% during laparoscopic cholecystectomy, 76% during neurosurgery in the sitting position, and 69–100% during laparoscopic hepatic resection [9–12]. Symptomatic gas emboli are however infrequent. There are unfortunately no firm incident data available. It is hardly possible to gain an incident from study data and there is no gas emboli register that could help compile data for statistical analysis. Hong et al. [13] interestingly found that incidence of venous gas embolism during robotic-assisted laparoscopic radical prostatectomy was 38% in comparison with 80% during radical retropubic prostatectomy. Paradoxical CO_2_ embolism, gas emboli in the arterial circulation, during laparoscopic surgery is most rare event. Arterial gas emboli can of course result in serious consequences such as neurologic injury. Systemic gas emboli are thought to be right to left shunting of venous gas embolism, either intracardiac due to patent foramen ovale (PFO) or extracardiac via transpulmonary air passage [14, 15]. Huang et al. [15] described a case report with presumed extracardiac paradoxical CO_2_ embolism, which resulted in neurologic deficit and weakness of all four limbs. In this case TEE showed acute gas embolism in both left and right side of the heart with no embolism detected on CT of MRI of the brain. We did not perform an acute TEE on our patient. TTE performed before the surgery did not however show any signs of left to right heart wall defects. No special maneuvers were performed in order to identify PFO (e.g., injecting contrast material into the bloodstream or applying positive pressure to the airway). Thus we cannot explicitly exclude the possibility that the patient has an undetected PFO. Extracardiac paradoxical gas embolism cannot be excluded either. The delay in onset makes gas emboli less likely. Carbon dioxide gas entrainment is generally seen only during gas insufflation and increase in pressure.

## 5. Migraine and Transitory Ischemic Attack

Migraine may be associated with neurology however uncommon. Our patient had no history of migraine. The headache was intense and bilateral; still a vascular “migraine” equivalent cannot be excluded. A first migraine attack in age of 50 associated with surgery seems however less likely. Transitory ischemic attack (TIA) generally defined as a neurologic deficit lasting less than 24 hours could be a plausible cause. Our patient had a BMI of 32, thus by definition being obese, but she had no history of cardiovascular disease or arthrosclerosis. CT-angiography showed no stenosis in her carotid arteries. One could speculate whether a relative cerebral ischemia following reversal of the head-down positioning during surgery and possibly mild hypotension following awakening and reduced stress may have caused a transient cerebral hypoperfusion and ischemia.

We are not able to provide any firm explanation to our patients' symptoms. The increased use of robotic surgery calls however for a vigilant awareness around side effects to avoid putting patients at risk. Maintenance of blood pressure, safe positioning, periodically checking, vigilance for the risk of CO_2_ emboli, cautious observation of EtCO_2_, and possibly added echocardiography in cases of suspicion is of great value to prevent possible complications.
